# Taiwanese Consumers’ Willingness to Pay for Broiler Welfare Improvement

**DOI:** 10.3390/ani9050231

**Published:** 2019-05-10

**Authors:** Yu-Chen Yang, Cheng-Yih Hong

**Affiliations:** 1 Department of Applied Economics, National Chung-Hsing University, Taichung 402, Taiwan; ycyang@dragon.nchu.edu.tw; 2 Department of Finance, Chaoyang University of Technology, Taichung 413, Taiwan

**Keywords:** farm animal welfare (FAW), willingness to pay, food safety concerns, ethical concerns, perceived consumer effectiveness, broiler

## Abstract

**Abstract:**

In this study, we explored the willingness to pay (WTP) for broilers raised under the high welfare system. The interval data model and the ordered probit model were used to investigate the factors that affect consumers’ WTP for broiler meat produced by farm animal welfare (FAW), practice. Our results from both methods suggest that socioeconomic characteristics such as education level, income level, gender, and age significantly affect consumers’ WTP. The food safety concerns of consumers and perceived consumer effectiveness also influence consumers’ WTP. Using the interval data method, we computed the mean and median of the estimated WTP from our survey sample. The mean was 46.7745 New Taiwanese dollar per kilogram. The marginal effects of the different variables are also presented.

**Simple Summary:**

In Taiwan, the development of farm animal welfare practice is in its beginning stage. Consumers’ attitude toward farm animal welfare products is important for the development of this practice. The main goal of this research is to explore the consumers’ willingness to pay for broilers’ welfare improvement and to identify the factors that affect this willingness to pay. The results of this study showed that consumers’ food safety concerns combined with farm animal welfare can influence consumers’ willingness to pay. The more consumers believed that they could make a difference in the improvement of animal welfare, the more they were willing to pay. Consumers who felt that farm animal welfare was the producers’ responsibility were less willing to pay. The results of this study can be used to evaluate whether or not farm animal welfare practice is market viable. Moreover, the results can be used to develop marketing strategies for high welfare broilers.

## 1. Introduction

In response to public pressure regarding farm animal welfare (FAW) in industrial livestock farming, the Brambell report claimed that farm animals are sentient beings capable of displaying fear, anger, and thirst. It inspired strong legislation to protect farm animals [1]. After that, the farm animal welfare council in the U.K. was established and proposed the concept of the Five Freedoms [2,3]. The Five Freedoms have gained global acceptance and commendation and have been incorporated in national regulations and food marketing schemes [4]. The ethical concerns associated with animal welfare are related to the animal’s quality of life [5,6].

Intensive animal farming has raised public concern from ethical, public health, environmental, and food safety perspectives [7,8,9,10,11,12]. Recently, in the European Union and the U.K., citizens have been paying more attention to the issue of farm animal welfare and these concerns have become a major force, pushing the government to implement higher standards [13,14].

Consumer attitudes toward FAW play an important role in promoting animal welfare practice. Without the support from consumers, the appropriate FAW standards cannot be implemented through the supply chain [15]. Generally, legislation on animal welfare originated as a result of pressure from the public. However, the animal welfare standards in food retail companies have a greater effect on animal welfare than do government regulations [3]. Consumer attitudes toward FAW products act as the main driving force behind food retail companies implementing appropriate FAW standards [16]. Like other agricultural products, livestock products need a marketing system to fulfill the final demand of consumers. A marketing system ranges from the slaughtering-house to food retailers, such as supermarkets. Therefore, we can say that the farm level demand for broilers is the derived demand from consumers demand for chicken. To attract consumers caring about FAW, supermarkets can use over-compliance with government regulation as a marketing strategy [3]. Under this circumstance, they will require their contract animal farm to comply with stricter standards than that promulgated by the government—a stricter FAW standard would be more effective in improving FAW than a relaxed one. For example, Carrefour in Taiwan will not accept eggs from production processes that do not comply with FAW standards, even though such a regulation has not been issued by the Taiwan government.

In Taiwan, the concept of the Five Freedoms has spread gradually among the public, and this is demonstrated by the increase in market share of farm animal welfare eggs from 1% to 7% between 2012 and 2018 [17]. From the results of this study, more than 77% of respondents were willing to pay more for high welfare broiler. The certification system in Taiwan for high welfare products is private. The system was established in 2007, providing a basic market segmentation of labeled and unlabeled goods. According to Taiwan’s society of agricultural standards, a third-party certification body, the maximum premium for high welfare products can be nine times larger than those for conventional products. This label system is guided by the principles of the Five Freedoms. So far, most of the farms certified by this label are shell eggs and indigenous chickens. For the broiler industry, only a few farms are able to fulfill the standards of this label system [18].

In order to improve the welfare of farm animals, it is important to create market segmentation and implement a market-driven strategy, particularly in countries where no legislation on high welfare standard is available. Improving the welfare of broilers will increase the cost of broiler production. If consumers are willing to pay the extra cost of high welfare broiler production, market segmentation should be applied, and a market-driven strategy should be implemented. However, if consumers do not want to pay the extra cost associated with high welfare broiler production, then government regulation is needed to improve the welfare of farm animals, and to prevent externality costs and other issues caused by intensive animal farming.

Retailers sponsoring the use of chicken and eggs produced under proper high welfare practice can appeal to more poultry farmers to participate in humane animal farming. The transformation of consumers’ ethical intentions into actual ethical buying serves as one of the driving forces influencing food retailers to develop appropriate high welfare standards. The gap between ethical intention and ethical buying remains a crucial issue [19].

Currently, in Taiwan, minimum animal welfare standards have not been passed to improve the welfare of farm animals. The opinion of food supply chain members and consumers’ perception toward the welfare of animals are important driving forces. For consumers, willingness to pay (WTP) is the amount of money a consumer wants to pay for achieving an attribute. In other words, WTP is the amount of money a consumer wants to give up in order to keep him in the same satisfaction status while obtaining an attribute [20]. The goal of this research is to investigate consumers’ willingness to pay (WTP) for broilers produced by high welfare practice and to determine the factors that affect this willingness to pay in Taiwan. The results of the study can be used to evaluate whether or not high welfare practice is market viable.

## 2. Materials and Methods

### 2.1. The Survey

We used a structured questionnaire to elicit information about consumers’ WTP for high welfare broiler meat in Taiwan. We employed a pilot survey before conducting the final survey. The survey period ranged from May to November 2018. Both online and hard copy surveys were distributed to respondents, with a total of 480 questionnaires returned to us. However, only 441 questionnaires were completed. During the survey, most of our respondents demonstrated a lack of familiarity with farm animal welfare issues. This lack of familiarity made it difficult for them to assign a financial value to high welfare products. In this study, convenient sampling was used. We sent our survey conductors to the exit of the Carrefour supermarket, which is one of the major channels that sell high welfare products in Taiwan. For the online survey, we selected respondents who frequently purchased high welfare or organic products. Generally, for both the online and hard copy survey, the Five Freedoms principle of high welfare was clearly explained on the cover page of our questionnaire. About 80% of people we selected rejected our invitation to answer the questionnaire.

The questionnaire contained three components. In the first section, we asked respondents about their purchasing habits and knowledge of high welfare products, including their purchasing concerns. Respondents’ food safety and ethical concerns in regard to high welfare products were also incorporated into this section. Moreover, the role of the government in supporting high welfare and perceived consumer effectiveness (PCE) regarding high welfare products were also contained in this part. For WTP, a list of options consisting of the various price intervals that respondents had to choose from was included in the second section. In the third section, respondents’ socioeconomics conditions were incorporated. The questionnaire didn’t reveal any personal information of the respondents so in this case ethical approval was not needed.

### 2.2. Variables

The variables included in the model to explain the WTP for high welfare broiler meat were categorized into three groups. The first group of variables consisted of perception variables, such as the respondent’s food safety concerns towards high welfare broiler meat, the respondent’s ethical perception towards broiler meat produced under high welfare practice, the respondent’s belief that they could make a difference, and the respondent’s belief that it was the producers’ responsibility to care about the welfare of farm animals that they keep. In previous literature, age has been suggested as one variable that can affect ethical consumption [21,22,23]. Besides age, the second group of factors included socioeconomic characteristics, such as education and income levels [24,25]. The third group consisted of other variables, such as whether or not the respondent had heard about high welfare practice, the concerns of the respondent while purchasing broiler meat, and the frequency of eating broiler meat (Table 1).

#### 2.2.1. Socioeconomic Conditions

In our survey, respondents were divided into five different groups based on their ages. However, none of the respondents who completed the questionnaires were older than 64. In the WTP equation, we added dummy variables for age. The dummy variables were denoted *ag4*, which represented the group of respondents aged between 45 and 55 years; and *ag5*, which represented the group of respondents aged between 55 and 64. The remaining respondents were incorporated into the reference group( For ag4 and ag5, the reference group covers respondents less than 45 years old). Gender was also incorporated, using a dummy variable (*gender* (Reference group is female)) set as 1 for male respondents and 0 for female ones. Regarding income, the dummy variable *inc4* was set as 1 for the group of respondents whose monthly incomes ranged between NTD 55000 and 70000; otherwise, it was set to 0. The dummy variable *inc5* represented the group of respondents with incomes more than NTD 70000 as 1; otherwise, *inc5* was set to 0. The remainder of the respondents were incorporated into the reference group. For respondents’ education, we used *ed4*, *ed5*, and *ed6* for respondents who had a College degree, Master’s degree, or Ph.D., respectively.

#### 2.2.2. Respondents’ Food Safety Concern

In the survey, respondents were asked about whether or not they agreed with the following statement: “The food safety quality can be improved if an animal is raised by farm animal welfare practice.” We used a Likert scale to measure the degree to which a respondent agreed with the statement. The Likert scale ranged from 1 to 5. If a respondent agreed strongly with the statement, the dummy variable, denoted *fs1*, was set to 1; otherwise, it was set to 0. If a respondent agreed with the statement, a dummy variable, denoted *fs2*, was set to 1; otherwise, it was set to 0. The reference group for dummy variables *fs1* and *fs2* represented the group that included respondents whose agreement scores for the statement were 3, 4, and 5.

#### 2.2.3. Respondents’ Ethical Concern

Similarly, in the survey, we used the degree to which a respondent agreed with the statement: “Paying courtesy to farm animals’ welfare is an appearance of the harmonious coexistence of people, animals, and the natural environment and is a symbol of a progressive society.” The extent to which respondents agreed ranged from 1 to 5. To measure respondent’s ethical concern [26], a dummy variable, eth1, was employed for the group of respondents who agreed very strongly with the statement. Similarly, a dummy variable, eth2, was employed for the group of respondents who agreed with the statement. The reference group for dummy variables eth1 and eth2 represented the groups that included respondents who scored 3, 4, and 5 in terms of agreement with the statement.

#### 2.2.4. Perceived Consumer Effectiveness

Perceived consumer effectiveness is an important factor that determines the transformation of ethical intention to ethical behavior [27]. In this study, we used the degree to which a respondent agreed with the following statement: “Your personal support for animal welfare can solve the problem of farm animals being abused” to measure the extent to which a respondent believes that his or her effort could make a difference. To measure respondent’s perceived consumer effectiveness, a dummy variable, denoted *pce1*, was used for the group of respondents who highly agreed with the statement. Similarly, a dummy variable, *pce2*, was used for the group of respondents who agreed with the statement. The reference group for dummy variables *pce1* and *pce2* is the group including the respondents whose agreement scores for the statement were 3, 4, and 5.

#### 2.2.5. Producers’ Responsibility

In this research, we used the degree to which a respondent agreed with the statement: “The industry should offer sufficient space for extension and appropriate facilities for farm animals to have an appropriate environment.” We used a dummy variable, denoted *prodh*, for the group of respondents whose agreement scores for the statement were 1, 2, and 3; otherwise, it was set to be 0. Therefore, the reference group included the group made up of respondents whose agreement scores for the statement were 4 and 5.

##### Research Methodology

To evaluate consumer WTP for broilers produced using high welfare practice in Taiwan, a contingent valuation survey was carried out to collect data. In this structured questionnaire, five WTP intervals (represented as percentage increase) were listed and respondents were asked to select the interval that they believed corresponded with their WTP. The intervals were numbered 1, 2, 3, 4, and 5, valued respectively as follows: (0–25%), (25–50%), (50%–75%), (75%–100%), and (100–∞%). The percentage increase was calculated as the increase in the price of broiler meat as a result of changing from conventional methods to high welfare methods, divided by the price of broilers produced under conventional methods. If the respondent’s WTP for high welfare broiler meat fell within Interval 1, then we considered the WTP to be very weak. If the WTP fell within Interval 2, we considered WTP to be weak. The WTP was considered moderate if it fell within Interval 3. Willingness to pay was considered strong if it fell within Interval 4, and willingness to pay was considered to be very strong if it fell within Interval 5. (Table 2).

We assumed that the willingness to pay (WTP) for high welfare broiler was expressed by the following equation:

(1) $$WTP^{*}=\sum_{i}\beta_{i}z_{i}+\varepsilon ,$$

where ε is assumed to be normally distributed with a mean of 0 and a variance $\sigma^{2},\;z_{i}$ are the explanatory variables. Willingness to pay ($WTP^{*}$) of consumers is unobservable.

We informed the respondents that the price of broiler chicken raised under the conventional farming method was 50 NTD/kg (NTD is new Taiwanese dollar). Accordingly, the corresponding monetary values for the various intervals were as follows: Interval 1, 0–12.5 NTD; Interval 2, 12.5–25 NTD; Interval 3, 25–37.5 NTD; Interval 4, 37.5–50 NTD; and Interval 5, 50–∞ NTD. The survey elicited both the strength and intervals of respondents’ willingness to pay for high welfare broilers. Therefore, both the ordered probit model and interval data method can be used. The factors that affect the WTP for high welfare broiler can be verified by both methods.

Unlike other contingent valuation methods that use bidding approaches, especially double bound dichotomous choices, this method allowed us to avoid the problem of bias resulting from changes in the incentive structure [28,29,30]. The interval data model includes more information and can improve the efficiency of estimation [31].

For this study, we denote an observable variable WTP. If Respondent i indicates that his willingness to pay is very weak, then WTP_i_ = 1; similarly, if the WTP is weak, then WTP_i_ = 2; if moderate, WTPi = 3; if strong, WTP_i_ = 4; and if very strong, WTP_i_ = 5.

The corresponding probability for WTP_i_ = j−1 is expressed as the following equation:

(2) $$\Phi (\gamma_{j}-\sum_{i}\beta_{i}z_{i})-\Phi (\gamma_{j-1}-\sum_{i}\beta_{i}z_{i}),$$

where $\gamma_{j}$ are unknown category threshold parameters that can be estimated, and Φ is the distribution function of standard normal distribution.

Therefore, Equation (2) can be used to estimate the value of the coefficient of $z_{i}$. Ordered qualitative response models were employed. The estimation results of $\beta_{i}$ cannot be used as the marginal impact of $z_{i}$. To estimate the marginal impact of $z_{i},$ we used the interval data method. In the interval data regression, the interval boundaries are known. Therefore, the probability that WTP^*^ falls into a specific interval ($\alpha_{j-1}$, $\alpha_{j}$) is F$\left(\frac{\alpha_{j}-\sum_{i}\beta_{i}z_{i}}{\sigma }\right)-\;F\left(\frac{\alpha_{j-1}-\sum_{i}\beta_{i}z_{i}}{\sigma }\right)$, where F(.) is the distribution function of the random variable ε in WTP^*^. If we assume the probability distribution of the random variable ε is normal, then the probability that the WTP^*^ falls into a specific interval ($\alpha_{j-1}$, $\alpha_{j}$) is

(3) $$\Phi \left(\frac{\alpha_{j}-\sum_{i}\beta_{i}z_{i}}{\sigma }\right)-\;\Phi \left(\frac{\alpha_{j-1}-\sum_{i}\beta_{i}z_{i}}{\sigma }\right).$$

## 3. Results and Discussion

### 3.1. Descriptions of Results from Two Methods

The empirical results from the ordered probit and interval data model were similar. The sign of the coefficients of variables were the same for both models, however, the explanations of the coefficients differed. The coefficients in the ordered probit model do not represent the marginal impacts of variables on the respondents’ WTP. The dependent variables in the ordered probit model were categorical ordinal variables. Hence, we could only evaluate the impact of variables on the probability of the occurrence of a specified category. Furthermore, the assumptions underlying the error term also differed. In the ordered probit model, the standard normal distribution was used. Four threshold parameters were estimated. In the interval data model, the random variables were normalized by assuming a scale parameter sigma (σ). Hence, only the coefficients in the interval data model could be used to represent the impact of the variables on WTP. From Table 3, it can be seen that out of 19 variables, 12 have coefficients that are significant. For a dummy variable, the coefficient represents the difference in the impact between respondents in a specific dummy group and those in the reference group. For a continuous variable, the coefficient represents its marginal impact.

### 3.2. Results from the Interval Data Method

From the econometric results of the interval data method, it can be seen that the coefficient of the gender variable is significant and negative. This implies that gender has a significant effect on WTP. The female respondents are more willing to pay for high welfare products. This result is consistent with previous literature. The coefficient of the variable, *nonp*, was significant and positive, which implies that a consumer who does not only consider price is willing to pay more for high welfare products. The coefficient of ag5 was positive and significant. This result implies that the group of respondents aged between 45 and 55 years old are more willing to pay than respondents in the reference group.

Regarding the respondents’ income, the coefficient of variable inc5 was both significant and positive. This implies that respondents in the group with incomes more than 70000 NTD are more willing to pay than respondents in the reference group. For education, the coefficients of ed4, ed5, and ed6 were all significant and positive. This result suggests that respondents with a College degree, Master’s degree, or Ph.D. are more willing to pay for high welfare products than respondents in the reference group.

Regarding consumers’ attitude toward high welfare products, the coefficients of variable *sf1* and *sf2* represent the differences in WTP between respondents in the dummy groups and the reference group. Both of the coefficients for variables *sf1* and *sf2* were significant and positive. This result suggests that respondents strongly agree with the statement that farm animal products are healthier and are inclined to pay more for high welfare products. Also, respondents strongly agreed with the statement that farm animal products were healthier and that they were inclined to pay more for these products. The results of this research indicate that consumer food safety concerns in regard to intensive farming significantly affect their WTP for high welfare products. The more a consumer agrees that high welfare products are healthier, the more he or she is willing to pay for high welfare products.

Regarding consumer’s ethical concerns, the coefficients of variables *eth1* and *eth2* represent the differences in WTP between respondents in the dummy groups and respondents in the reference group. Both of the coefficients for variables *eth1* and *eth2* were insignificant but positive. This indicates that consumer’s ethical concern of high welfare has no impact on their WTP for high welfare products.

The coefficient of variable *prodh* represents the difference in WTP between respondents who agreed with the statement that producers are responsible for animal welfare and should provide a decent environment for farm animals, and those who did not believe so. The coefficient of variable *prodh* was significant and negative. This result implies that respondents who agree that producers are responsible for animal welfare are less willing to pay for high welfare products.

The coefficient of variable *pce1* represents the difference in WTP between respondents who strongly believe that they can have an effect in improving the welfare of farm animals and those who believe that they can have no effect. The coefficient of variable *pce1* was significant and positive. This implies that a respondent who strongly believes that he or she can have an effect in improving the welfare of farm animals is more willing to pay for high welfare products than one who believes that he or she has no effect. The result was similar for variable *pce2*—with a coefficient that was both significant and positive.

After estimating the coefficients of variables in Equation (1), we were able to estimate the WTP for respondents with varying socioeconomic characteristics and varying degrees of agreement in terms of the link between food safety, ethical quality, and high welfare broiler products. By substituting the coefficients in Equation (1) with the estimations of the coefficients in Table 3 from the interval data model, we obtained the sample mean and median of the estimated WTP for high welfare broiler meat in Taiwan. The estimates of the WTP are shown in Table 4. The sample mean of consumer WTP was 28.0648 NTD/kg, while the sample median of WTP was 28.4762 NTD/kg.

### 3.3. Results from the Ordered Probit Model

Since the meaning of the coefficients for the variables in the ordered probit model differed from those in the interval data model, it cannot represent the marginal impact. We computed changes in the probability of a specific category outcome occurring. In the ordered probit model, we used the following equation:

(4) $$\frac{\Delta \mathrm{Pr}\left(WTP=J-1\right)}{\Delta x_{i}}=\Phi \left(WTP=J-1|x_{i}=1\right)-\Phi \left(WTP=J-1|x_{i}=0\right).$$

The marginal effects are shown in Table 5. It can be seen that males are more likely to have lower WTP compared to females. The probability that categories specify very strong, strong, and moderate groups both diminish. However, the probabilities for weak and very weak groups increase. This result implies that, in general, female respondents have a higher likelihood of willingness to pay more.

For age, the marginal impact of age5 was negative for very weak WTP and weak WTP. The marginal effects were positive for modest, strong, and very strong WTP. This implies that respondents aged between 55 and 65 years had a higher probability of modest, strong, and very strong WTP compared with those in the reference group.

For education levels, in comparison with those in the reference group, respondents with a College degree, Master’s degree, or Ph.D. had a higher probability of having modest, strong, and very strong WTP. For weak and very weak WTP, the marginal effects were negative.

The marginal effects of *fs1* were negative for very weak WTP and weak WTP. For modest, strong, and very strong WTP, the marginal effects were positive. This indicates that a consumer who had very strong recognition that high welfare products are healthier had a higher probability of having modest, strong, and very strong WTP compared with a consumer in the reference group. For weak and very weak WTP, the marginal effects were negative. The marginal effects of *fs2* on each of the categories were similar.

Regarding perceived consumers effectiveness, the marginal impacts of *pce1* were negative for outcomes of weak and very weak WTP; however, for outcomes of strong and very strong WTP, marginal effects were all positive. Respondents who had a very strong belief that their contribution could make a difference in improving the welfare of farm animals had a higher likelihood to have modest, strong, and very strong WTP than those in the reference group.

For consumers’ purchasing concern for broiler, the marginal effect of *nonp* was negative for outcomes of weak and very weak WTP; and was positive for outcomes of modest, strong, and very strong WTP. This indicates that if respondents did not only care about pricing, they were more likely to pay more in comparison with those who only cared about broiler meat price.

Concerning consumers’ altitude toward producers’ responsibility for high welfare, the marginal effect of *prodh* was positive for outcomes of weak and very weak WTP; and was negative for those of modest, strong, and very strong WTP. This result implies that respondents who agree that it is the responsibility of the farmer to provide a decent environment for farm animals have a higher likelihood to have weak and very weak WTP than those who do not.

### 3.4. Marketing Strategies for High Welfare Products

The results of this research demonstrate that if consumers recognize that animal welfare products are healthier, they are willing to pay premium prices for high welfare products. Such a result implies that adopting high welfare practices will not result in businesses losing competitive advantage due to increased production costs [32]. Producers adopting high welfare methods could gain competitive advantage as a result of market segmentation by providing healthier products to consumers.

Food safety concerns over intensive animal farming practices are the driving force for consumers to purchase ethically products produced by high welfare methods. Consequently, in order to provide consumers more links to high welfare practices, one suggested strategy is to assist consumers to completely comprehend the ethical high welfare production processes. Additionally, another strategy is helping consumers realize that their purchasing of high welfare products is beneficial. The payoff for ethical buying is the improvement in consumers’ food safety.

Regarding perceived consumer effectiveness (PCE), our results demonstrate that PCE is one factor that can inspire consumers’ buying intentions [33,34,35,36]. Therefore, in order to encourage consumers’ intention to buy high welfare products, their PCE should be motivated. A number of researchers have discussed how to activate consumer PCE [37,38,39]. Accumulation of knowledge in high welfare practices and awareness of individuals’ effort will make a difference in solving the problem of weak consumer PCE. Therefore, animal welfare education should begin from the early childhood stage. In addition, government and non-profit organizations should campaign on behalf of improved and humane animal welfare practice to demonstrate to individual consumers that they are not alone.

### 3.5. The Gap between Ethical Intention and Ethical Buying

Our results reveal that some consumers with high ethical concerns for farm animal welfare issues are not actually willing to pay the premium for high welfare products. There exists a gap between ethical concern and ethical buying. This attitude–behavior gap is an example of so-called consumer dualism, discussed by Verbeke (2009) and Grunert (2006) [40,41]. One factor possibly discouraging customers from participating in dollar voting is their lack of trust in the ability of producers to fulfill all the requirements of high welfare. Therefore, they feel that their efforts in that regard are impractical. This ambiguity may also result from the fact that consumers want to support high welfare practice, but they worry that the price is too high. As mentioned earlier, farm animal welfare is deemed a public good. This is a problem related to the phenomenon of free riding [42,43,44].

Consumers who think that producers are responsible for high welfare are less willing to pay for farm animal products. This result could serve as an explanation of the gap between ethical concern and ethical buying. Consumers are concerned with the issue of animal welfare; however, they feel disengaged from animal welfare issues and believe that it is not their personal responsibility.

## 4. Conclusions and Limitations of Research

One relevant result emerging from this study is the agreement between interval data methods and the ordered probit method. In addition, the results of this study indicated that consumers’ food safety concern regarding high welfare was one factor that influenced consumers’ WTP for high welfare broiler chicken meat. Socioeconomic characteristics such as education, income level, gender, and age also influence consumers’ willingness to pay. Females were more willing to pay than males. Consumers with a College degree, Master’s degree, or Ph.D. were more willing to pay compared with those with an educational attainment below the college level. Consumers with monthly income levels ranging between 55000 and 700000NTD were also more willing to pay than the others. Respondents who fell within the age range of 55‒65 were more willing to pay than others. Regarding respondents perceived consumer effectiveness, our results demonstrated that the more consumers believe that they could make a difference in solving the animal welfare problem; the more they were willing to pay for high welfare products. However, consumers who felt that farm animal welfare was the producers’ responsibility were less willing to pay.

Although the survey question should be more concise and less subjective, the results of this study showed that an individual’s moral intensity is irrelevant to WTP for improvement in broiler welfare.

During the survey, some respondents felt that it was hard to assign a financial value to the broiler chicken produced by high welfare practice. This may be one of the reasons why some respondents gave up answering the questionnaire completely.

## Figures and Tables

**Table 1:** Descriptive Statistics.

Variable	Definition	Mean	Std. Dev
heard	Respondents who have heard about farm animal welfare	0.421769	0.494403
male	Respondents who are male	0.462585	0.499164
eth1	Respondents who very strongly agree that high welfare practice is ethical	0.492064	0.500505
eth2	Respondents who strongly agree that high welfare practice is ethical	0.328798	0.47031
fs1	Respondents who very strongly agree that products produced by high welfare practice are healthier	0.560091	0.49694
fs2	Respondents who strongly agree that products produced by high welfare practice are healthier	0.285714	0.452267
pce1	Respondents whose belief that they can make a difference in solving animal welfare problem is very strong	0.253968	0.435774
pce2	Respondents whose belief that they can make a difference in solving animal welfare problem is strong	0.356009	0.479362
ag5	Respondents’ age is between 55 and 65 years old	0.102041	0.303046
ag6	Respondents’ age is between 65 and 75 years old	0.040816	0.198089
ed4	Respondents have College degree	0.544218	0.498607
ed5	Respondents have Master’s degree	0.14966	0.357143
ed6	Respondents have Ph.D.	0.036281	0.187201
inc4	Respondents’ income is between 55000–70000 NTD	0.090703	0.287512
inc5	Respondents’ income is more than 70000 NTD	0.131519	0.338351
nonp	Price is not the respondents’ only concern	0.950113	0.217958
freq_c	Frequency of eating chicken meat	3.011338	1.601805
prodh	Respondents think that farm animal welfare is the responsibility of producers	0.938776	0.240014

**Table 2:** Strength of willingness to pay for broilers produced by high welfare practice.

Strength of Willingness to Pay	Interval Label
Between 0 and 25 percent (Very weak)	Interval 1
Between 25 and 50 percent (Weak)	Interval 2
Between 50 and 75 percent (Modest)	Interval 3
Between 75 and 100 percent (Strong)	Interval 4
More than 100 percent (Very Strong)	Interval 5

**Table 3:** Estimation Results.

Variables	Interval Data Regression	Ordered Probit Model
heard	1.6354	0.1521
male	−2.745^**^	−0.2418^**^
eth1	2.4394	0.2128
eth2	−3.0056	−0.2591
fs1	5.5652^*^	0.5332^*^
fs2	5.3528^*^	0.5088^*^
pce1	5.0128^***^	0.4408^***^
pce2	1.0305	0.08418
ag5	3.9727^*^	0.3570^*^
ag6	0.2342	−0.006
ed4	3.4320^**^	0.3081^**^
ed5	7.2680^***^	0.6344^***^
ed6	7.6075^**^	0.6584^**^
inc4	2.4710	0.2259
inc5	6.7881^***^	0.5937^***^
nonp	4.8749^*^	0.4751^*^
freq_c	0.5389	0.0486
Prodh	−8.8927^***^	−0.8176^***^
Constant	19.4765^***^	
/lnsigma	2.4357^***^	
sigma	11.4243	
threshold parameter1		−0.3368
threshold parameter 2		0.4404
threshold parameter3		1.7015
threshold parameter 4		2.6773
	$\chi^{2}=128.86$ *p*-value=0	$\chi^{2}=127.45$ *p*-value=0

* is significant at 10% confidence level. ** is significant at 5% confidence level. *** is significant at 1% confidence level.

**Table 4:** Estimated willingness to pay for broiler produced by high welfare practice.

	Mean	Maximum	Median	Minimum
Willingness to pay	46.7745	81.0495	47.4603	16.6580

**Table 5:** Marginal effects.

Variables	Very Weak	Weak	Moderate	Strong	Very Strong
heard	−0.0322	−0.0168	0.0103	0.02400	0.0147
male	0.0512^**^	0.0267^**^	−0.0163^**^	−0.0381^**^	−0.0234^**^
eth1	−0.0450	−0.0235	0.01437	0.0336	0.0206
eth2	0.0548	0.0286	−0.0175	−0.0409	−0.0250
fs1	−0.1128^*^	−0.0588^*^	0.0360^*^	0.0841^*^	0.0515^*^
fs2	−0.1077^*^	−0.0561^*^	0.0344^*^	0.0803^*^	0.0492^*^
pce1	−0.0933^***^	−0.0486^***^	0.0298^***^	0.0695^***^	0.0426^***^
pce2	−0.0178	−0.0093	0.0057	0.0133	0.008
age5	−0.0756^*^	−0.0394^*^	0.0241^*^	0.0563^*^	0.0345^*^
age6	0.0014	0.0007	−0.0004	−0.0010	−0.0006
ed4	−0.0652^**^	−0.0340^**^	0.02081^**^	0.0486^**^	0.0298^**^
ed5	−0.1342^***^	−0.0700^***^	0.0428^***^	0.1001^***^	0.0613^***^
ed6	−0.1393^**^	−0.0726^**^	0.0444^**^	0.10381^**^	0.0636^**^
in4	−0.0478	−0.0249	0.01525	0.03561	0.0218
in5	−0.1256^***^	−0.0655^***^	0.040^0**^*	0.09361^***^	0.0574^***^
nonp	−0.1005^**^	−0.0524^**^	0.0321^**^	0.0749^**^	0.0459^**^
freq_c	−0.0103	−0.0054	0.0033	0.0077	0.0047
peodh	0.1723^***^	0.0902^***^	−0.0552^***^	−0.1290^***^	−0.0790^***^

* is significant at 10% confidence level. ** is significant at 5% confidence level. *** is significant at 1% confidence level.
